# Three Ureters Demonstrated during Life; Ureter-Catheterization Giving Three Different Urines, One Infected with Gonococci

**Published:** 1907-01

**Authors:** 


### THREE URETERS DEMONSTRATED DURING LIFE; URETER-CATHETERIZATION GIVING THREE DIFFERENT URINES, ONE INFECTED WITH GONOCOCCI.

---

Dr. Bransford Lewis, (*Medical Record,* October 6th, 1906) reports the history of this very interesting case at length. A diagnosis was made of the three ureters by an examination through his rectangular cystoscope. In this article he says:

"On inserting the rectangular cystoscope into the bladder and taking a preliminary survey, I was surprised to observe what appeared to be two ureter openings to the left of the median line, on the line of the interureteric fold, about three-sixteenths of an inch apart, while another, single, ureter opening showed in the usual location on the right side. The three ureter openings thus seen were readily recognized by others present at the examination, Drs. Burford, Wolff, and Scharff). We supposed that the two openings on the left side were simply the duplicated outlet of a bifurcated ureter.

"The right angle periscope was then replaced by the double-catheterizing part, armed with two catheters; a catheter was easily introduced into each of the two openings of the left side, passing upwards for what seemed about the natural distance to reach the kidney. They could not be felt to touch one another in transit. The ureter on the right side was left for future investigation, as my catheterizing attachment only provided for catheterizing two ureters at a time.

"When the cystoscope was withdrawn, leaving the two catheters in the openings on the left side, prompt drainage was established from both, the one draining the extreme opening, however, being much more active than its companion. 'The urines coming from the two catheters displayed not the slightest indication of having any relation to one another. One would drain regularly, after its own rhythm, while the other would follow its own periodicity quite out of harmony with the first. The extreme left one drained about three times as rapidly as the middle one. The urine coming from the extreme left opening was of much lighter specific gravity (1.005) than that from the middle (1.010); it was much lighter in color, was quite cloudy, with flocculi floating in it; while the other was clear and yellow, more like normal urine. On microscopical examination the left ureter urine showed much pus and epithelium and a moderate number of blood cells, and contained in quite a number of the pus cells were colonies of diplococci that gave the customary appearance of gonococci to the positive methylene blue stain, and decolorized after the Gram stain. In the urine from the middle opening, no pus cells and no organisms were found."

In this case, too, he utilized the x ray to confirm the diagnosis, and was able to demonstrate this as is shown by the following description:

"Three days afterward, February 27th, I carried out the excellent plan suggested by Kolischer and Schmidt for identifying the ureters throughout their course by means of lead fuse-wire filling the ureter catheters that are passed up to the kidneys, and made evident by an x ray photograph. Three catheters, armed with wire, were successfully introduced into the three ureter openings and pushed up toward the renal regions, after which two x ray photographs were taken by Dr. R. D. Carman, with very satisfactory results. This skiagraph makes it evident that the ureter on the right side of the body has no connection with the two on the left side. Those of the left side, after leaving their respective openings at the angle of the trigone run parallel with one another in following the direction of the pelvic hexagon, and ascend in the manner usually followed by the left ureter."—*St. Louis Medical Review,* Dec. 8th, 1906.

